# Genetic Relationship between Cocirculating Human Enteroviruses Species C

**DOI:** 10.1371/journal.pone.0024823

**Published:** 2011-09-12

**Authors:** Maël Bessaud, Marie-Line Joffret, Barbara Holmblat, Richter Razafindratsimandresy, Francis Delpeyroux

**Affiliations:** 1 Institut Pasteur, Unité Postulante de Biologie des Virus Entériques, Paris, France; 2 INSERM U994, Paris, France; 3 Institut Pasteur de Madagascar, Unité de Virologie, Antananarivo, Madagascar; Johns Hopkins School of Public Health, United States of America

## Abstract

Recombination events between human enteroviruses (HEV) are known to occur frequently and to participate in the evolution of these viruses. In a previous study, we reported the isolation of a panel of viruses belonging to the *Human enterovirus species C* (HEV-C) that had been cocirculating in a small geographic area of Madagascar in 2002. This panel included type 2 vaccine-derived polioviruses (PV) that had caused several cases of acute flaccid paralysis in humans. Previous partial sequencing of the genome of these HEV-C isolates revealed considerable genetic diversity, mostly due to recombination. In the work presented herein, we carried out a more detailed characterization of the genomes of viruses from this collection. First, we determined the full VP1 sequence of 41 of these isolates of different types. These sequences were compared with those of HEV-C isolates obtained from other countries or in other contexts. The sequences of the Madagascan isolates of a given type formed specific clusters clearly differentiated from those formed by other strains of the same type isolated elsewhere. Second, we sequenced the entire genome of 10 viruses representing most of the lineages present in this panel. All but one of the genomes appeared to be mosaic assemblies of different genomic fragments generated by intra- and intertypic recombination. The location of the breakpoints suggested potential preferred genomic regions for recombination. Our results also suggest that recombination between type HEV-99 and other HEV-C may be quite rare. This first exhaustive genomic analysis of a panel of non-PV HEV-C cocirculating in a small human population highlights the high frequency of inter and intra-typic genetic recombination, constituting a widespread mechanism of genetic plasticity and continually shifting the HEV-C biodiversity.

## Introduction

The members of the *Human enterovirus species C* (HEV-C), genus *Enterovirus*, family *Picornaviridae*, are non-enveloped viruses with a single positively-stranded RNA genome. The virions contain one copy of the genome, which is about 7,500 nucleotides (nt) in length and consists of two untranslated regions (5′- and 3′-UTR) bordering a unique large open reading frame. The encoded polyprotein is first cleaved into three precursors (P1 to P3) subsequently cleaved into functional proteins. P1 gives rise to the four structural capsid proteins (VP1 to VP4); P2 and P3 generate non-structural proteins involved in the viral cycle [1]. Currently, 20 serotypes have been identified, including the three serotypes of poliovirus (PV-1 to -3) that can induce severe and potentially fatal cases of poliomyelitis in humans [2], [3].

The evolution of HEV is driven by mutations due to the proneness to error of the viral RNA polymerase and frequent recombination events between the genomes of different viruses infecting the same cell [4], [5], [6], [7]. The three vaccinal strains of PV (called Sabin strains) may also engage in recombination, thereby promoting the emergence of pathogenic vaccine-derived PV (VDPV), which have been implicated in many poliomyelitis outbreaks [8], [9], [10]. Most of these VDPV have a chimeric genome combining mutated capsidic sequences from Sabin strains with non-structural sequences from non-PV HEV-C [11].

Genetic exchanges between PV and other HEV-C have been widely documented [12], [13], [14], [15], [16], [17], [18], [19] but those between non-PV HEV-C remain poorly described.

In previous works [14], [20], we reported the characterization of type 2 VDPV responsible for 4 human cases of acute flaccid paralysis in humans in the south-western part of Madagascar (Tolagnaro district) in 2002. Investigations of healthy children to identify the enteroviruses circulating in the small area in which the poliomyelitis cases occurred revealed the presence of highly diverse HEV-C genomes in this region, with a high frequency of co-infections with viruses of different types. We collected about 300 stool samples, 22% of which tested positive for non-PV HEV, with 80% of the HEV isolates identified as HEV-C belonging to 5 different types [14]. Partial sequencing of the HEV-C genomes in different genomic regions highlighted the occurrence of intertypic recombination. Some of the recombinant genomes displayed genomic sequences closely related to non-PV sequences present in VDPV, consistent with recombination between PV and non-PV HEV-C.

This dense and diverse HEV-C ecosystem constitutes a valuable tool for elucidating the processes shaping enteroviral biodiversity.

In the present work, we investigated the molecular characteristics of the cocirculating Madagascan HEV-C isolates in more detail.

First, we analyzed the phylogenetic relationships between Madagascan non-PV HEV-C isolated in 2002 and HEV-C isolated in other contexts. We determined the full VP1 sequences of 41 HEV-C isolates obtained in Madagascar in 2002 and available in our laboratory. We found that the Madagascan isolates had molecular specificities that differentiated them from other strains isolated elsewhere.

Second, we investigated the genetic relationships between these isolates by determining the full-length genomes of 10 HEV-C from different lineages. We carried out phylogenetic and recombination analyses on these sequences and determined the breakpoint locations. Most HEV-C isolates showed a mosaic genome resulting from multiple intra- and intertypic recombination events; our results the importance of genetic recombination as a widespread mechanism of viral genetic plasticity in this HEV-C ecosystem.

## Materials and Methods

### Virus isolates

Non-PV HEV-C viruses were isolated in Madasgascar in June 2002 [14], [21]. All work with infectious viruses was carried out in a BSL-2 facility. The viruses were grown in HEp-2c monolayers in DMEM supplemented with 2% foetal calf serum and 2 mM*_L_*-glutamine at 37°C. Before sequencing, each field isolate was purified twice by the limiting dilution method in 96-well dishes and then passaged once in a T-25 flask. Viral RNA was extracted with the High Pure Viral RNA kit (Roche Diagnostics).

### Nucleotide sequencing

The sequences of the field viruses isolated in Madagascar in 2001-2002 had already been partly determined [14], [21]. The complete VP1 sequences of 41 of these isolates were obtained by using both degenerated [22] and virus-specific primers and submitted to GenBank (accession numbers are indicated in Table 1).

**Table 1:** Molecular typing based on full-length VP1 sequences.

Field isolates	Closest prototype strain	VP1 nucleotic homology	VP1 peptidic homology	Accession number
66122	CV-A11 G9	82.4%	96.7%	JF260917
66989	CV-A11 G9	82.1%	97.4%	AM779132
66990	CV-A11 G9	81.7%	97.0%	JF260918
67004	CV-A11 G9	81.9%	97.1%	AM779134
67874	CV-A11 G9	84.0%	98.0%	JF260919
68101	CV-A11 G9	84.1%	98.0%	AM779138
68123	CV-A11 G9	82.5%	96.7%	AM779135
68128	CV-A11 G9	84.0%	97.7%	AM779155
68140	CV-A11 G9	83.8%	97.4%	AM779140
68226	CV-A11 G9	84.0%	98.0%	AM779139
65965	CV-A13 Flores	77.0%	91.3%	AM779118
66025	CV-A13 Flores	77.1%	91.3%	AM779119
66114	CV-A13 Flores	76.8%	89.6%	AM779126
66119	CV-A13 Flores	76.7%	90.3%	AM779127
66125	CV-A13 Flores	76.3%	89.6%	AM779125
66998	CV-A13 Flores	77.2%	91.9%	AM779121
67001	CV-A13 Flores	77.0%	90.9%	JF260920
67598	CV-A13 Flores	76.9%	91.6%	AM779120
67900	CV-A13 Flores	75.1%	90.9%	JF260921
68095	CV-A13 Flores	77.8%	91.6%	JF260922
68145	CV-A13 Flores	76.6%	91.3%	JF260923
68244	CV-A13 Flores	74.2%	90.9%	AM779130
67610	CV-A17 G-12	78.2%	93.8%	JF260924
68088	CV-A17 G-12	76.3%	91.8%	AM779115
68094	CV-A17 G-12	76.7%	92.8%	AM779136
68125	CV-A17 G-12	76.2%	91.8%	AM779114
68138	CV-A17 G-12	77.0%	91.8%	AM779112
68146	CV-A17 G-12	76.7%	91.8%	AM779111
68154	CV-A17 G-12	77.3%	93.1%	JF260925
65902	CV-A24 EH24	79.8%	92.1%	AM779154
67897	CV-A24 DN19	77.0%	92.5%	AM779149
67602	HEV-99 10636	79.2%	93.1%	AM779151
67877	HEV-99 10636	79.1%	91.4%	AM779148
67890	HEV-99 10636	79.7%	93.1%	AM779150
68086	HEV-99 10636	79.2%	92.1%	AM779145
68098	HEV-99 10636	79.3%	91.8%	AM779156
68118	HEV-99 10636	79.5%	92.4%	AM779142
68150	HEV-99 10636	79.7%	92.7%	AM779144
68152	HEV-99 10636	79.6%	92.4%	AM779146
68229	HEV-99 10636	78.9%	91.1%	JF260926
68269	HEV-99 10636	78.6%	93.1%	AM779153

Full-length sequences of 10 viruses were determined by gene-walking. The sequences of the 5′ genome ends were determined by using the 5′/3′ RACE kit (Roche Diagnostics) following the manufacturer's instructions. Sequencing was conducted with the BigDye terminator v3.1 kit (Applied Biosystems) and an ABI Prism 3140 automated sequencer (Applied Biosystem). Full-length genomic sequences were obtained by assembling sequencing electropherograms with CodonCode Aligner version 2 (CodonCode Corporation) and were submitted to GenBank (accession numbers JF260917-JF260926).

### Sequence analyses

The sequences of the prototype viruses coxsackievirus (CV) A11 strains G-9 and Belgium-1, CV-A13 strains G-13 and Flores, CV-A17 strain G-12, CV-A24 strains Joseph, DN-19 and EH24, HEV-99 strain GA84-10636, PV-2 strain Sabin and PV-3 strain Sabin (referred to as Sabin 2 and Sabin 3 throughout the text), CV-A10 strain Kowalik and echovirus (EV) -19 strain Burke were retrieved from the GenBank database (accession numbers AF499638, AF499636, AF499640, AF499637, AF499639, EF026081, AF081347, D90457, EF555644, AY184220, K00043, AY421767 and AY302544, respectively).

Full-length sequences of the CV-A17 strain 67591 and the PV-2 strain MAD004 had been previously reported (accession numbers FM955278 and AM084223, respectively).

**Figure 1:**
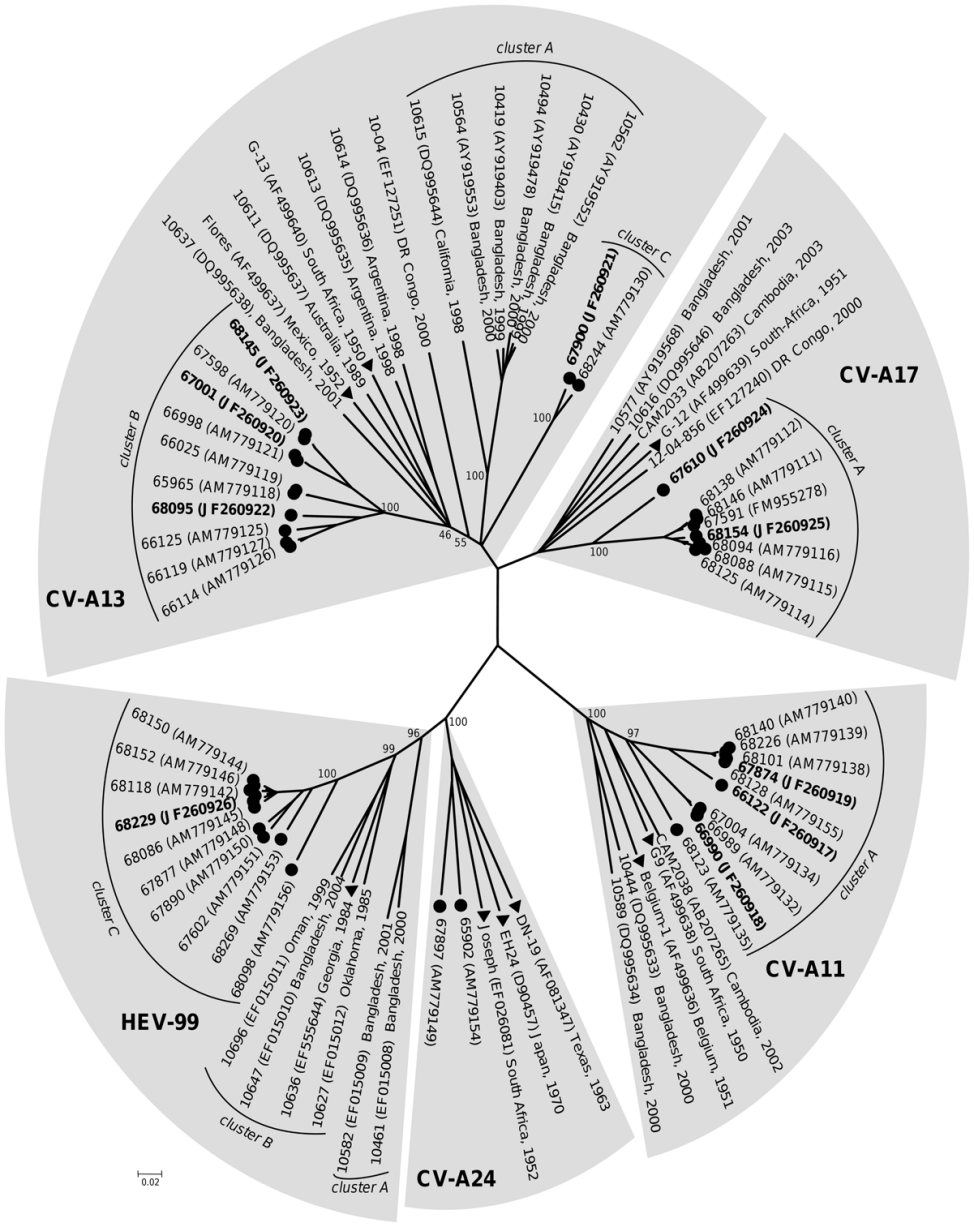
Phylogenetic relationships between Madagascan HEV-C field isolates collected in 2002 and other isolates for which sequences are available in GenBank, based on full-length VP1 sequences. The length of the branches is proportional to the number of nucleotide changes (percent divergence). The percentage of bootstrap replicates is indicated for the main nodes. Each area of grey shading corresponds to a serotype. The field strains isolated in Madagascar in 2002 are indicated by circles; the isolates whose full-length genome was subsequently sequenced are indicated in bold. For the other isolates, the location and year of isolation are indicated in the tree. Triangles indicate the prototype strains.

**Figure 2:**
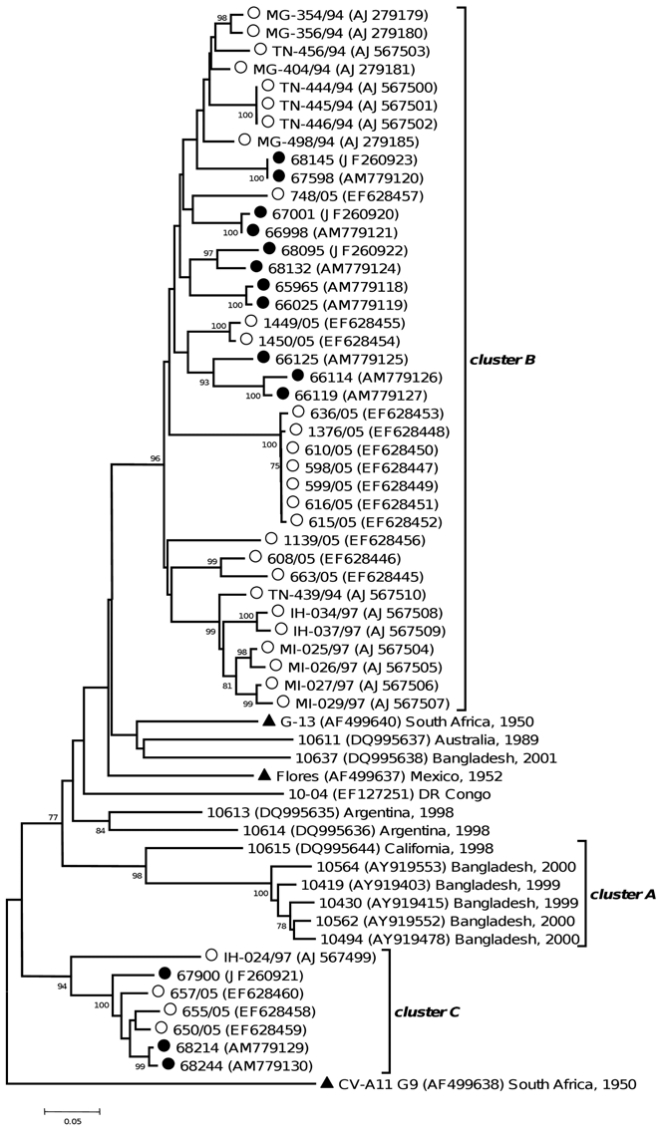
Phylogenetic relationships between Madagascan CV-A13 field strains and other CV-A13 isolates for which sequences are available in GenBank, based on 3′ one-third of the VP1 region (∼300 nt). The length of the branches is proportional to the number of nucleotide changes (percent divergence). The percentage of bootstrap replicates is indicated if higher than 70%. The field strains isolated in Madagascar are indicated by full circles if isolated in 2002, by open circles if isolated in other years. For the other isolates, the location and year of isolation are indicated in the tree. Triangles indicate the prototype strains. The CV-A11 G9 sequence was introduced for correct rooting of the tree.

Full or partial VP1 sequences of CV-A11, CV-A13, CV-A17 and HEV-99 clinical isolates were retrieved from GenBank (see Figures 1 and 2 for the corresponding accession numbers).

Multiple sequence alignments were performed with CLC Main Workbench 6.0 software (CLC bio). Phylograms were constructed with the MEGA 4 program [23] using the Jukes-Cantor algorithm for genetic distance determination and the Neighbor-Joining method. The robustness of the resulting trees was assessed with 1,000 bootstrap replications.

Recombination was analyzed with the Recombination Detection Program v.3.22 [24] and the SimPlot software [25]. Levels of pairwise identities between sequences were determined by using the Hamming distance method [26] and manual bootscanning was carried out with the Kimura distance model [27] with a 200 nt-wide window and a step size of 20 nt.

## Results

### Molecular typing

The most common method for the molecular typing of HEV field strains is based on comparison of their VP1 sequences with those of prototype strains [22], [28], [29], [30], [31].

The Madagascan isolates collected in 2002 had already been typed by taking into account the ∼300 nt-long 3′ one-third of VP1 [14]. However, this region is sometimes too short for the accurate typing of field strains [32]. We therefore refined our typing results by obtaining full-length VP1 sequences for 41 HEV-C isolates.

Twenty-nine isolates had been classified as CV-A11, CV-A13 and CV-A17 on the basis of their partial VP1 sequences. For all these isolates, typing based on their respective full-length VP1 sequence confirmed our previous results. Each virus displayed with the prototype strain of its type a VP1 amino-acid (aa) identity higher than 88.0% (Table 1), which is considered to be the cut-off value allowing an unambiguous typing [32].

The 12 remaining isolates had been first identified as CV-A24 [14]. However, only 2 of them (65902 and 67897) featured an aa identity higher than 88.0% with the CV-A24 prototype strains (Table 1).

The 10 other isolates displayed with the CV-A24 prototype strains an aa identity ranging from 83.6% to 84.9% in the VP1 region. These isolates were more similar to the strain 10636, which is the prototype of the recently described type HEV-99 [32], with a VP1 aa identity ranging from 91.1% to 93.1% (Table 1). These 10 isolates were therefore reclassified as HEV-99.

### Molecular epidemiology of Madagascar HEV-C isolates

We evaluated the relationships between the HEV-C strains circulating in Madagascar in 2002 and those isolated in other contexts by aligning the VP1 regions of the 41 Madagascan isolates with those of clinical isolates and prototype strains available in GenBank [32], [33], [34] and using phylogenetic tools. Figure 1 displays the phylogram obtained from this nucleotidic alignment. The tree drawn from the deduced peptidic alignement displayed a similar pattern (data not shown).

All the 15 CV-A11 VP1 sequences, including those of the two prototypes, were very close to each other (nt identity ≥77.7%, aa identity ≥95.1%). The 10 Madagascan isolates clustered together (bootstrap value of 97%) and displayed a low range of diversity, featuring together a nt identity ≥83.8% and an aa identity ≥96.7%.

The CV-A17 VP1 sequences were all very similar (nt identity ≥75.5%; aa identity ≥91.8%). All together, the Madagascan CV-A17 sequences defined a single cluster (bootstrap value of 100%). Seven of them were very close to each other (nt identity ≥94.0%; aa identity ≥95.1%); while slightly divergent, the 67610 sequence remained relatively close to the other sequences of this cluster (nt identity ≥84.5%; aa identity ≥92.8%).

The sequences of clinical strains isolated in several countries in the 2000's and those of the prototype strain G-12 isolated in 1951 displayed together a nt identity ≥77.9% and an aa identity ≥93.0%; they did not group together into a single cluster.

As reported above, 10 isolates initially identified as CV-A24 actually belonged to the HEV-99 type. All the HEV-99 VP1 sequences were closely related (nt identity ≥72.8%; aa identity ≥87.5%). In this type, the VP1 sequences grouped into three different clusters supported by high bootstrap values (96%, 99% and 100%, respectively). All the Madagascan sequences fell in cluster C, which contained no other sequences. The sequences of this cluster displayed a nt identity ≥85.8% and an aa identity ≥94.1%.

Unlike the VP1 sequences of the other HEV-C types considered above, the CV-A13 VP1 sequences displayed a wide diversity with an overall identity ≥69.7% and ≥84.5% at nucleotidic and peptidic level, respectively.

As previously reported [32], the CV-A13 sequences fell into several clusters.

Several sequences of viruses isolated in Bangladesh and California defined cluster A, which was supported by a bootstrap value of 100%. In this cluster, the nt identity was ≥79.9% and the aa identity ≥93.0%.

Most of the Madagascan CV-A13 sequences (10 of 12) constituted a unique cluster (cluster B, bootstrap value of 100%) that contained no other sequences. The identity in this cluster was ≥86.3% and ≥93.0% at nucleotidic and peptidic level, respectively.

The VP1 sequences of isolates 67900 and 68224 featured together a nt identity of 92.9% and an aa identity of 97.1%. These two sequences significantly differed from the other CV-A13 ones (nt identity ranging 70.3% to 75.3% and an aa identity ranging from 87.1% to 91.3%) and formed a third cluster (cluster C) supported by a bootstrap value of 99%.

The seven remaining CV-A13 sequences, including those of the two prototype strains G-13 and Flores, did not form a well defined cluster (most bootstrap values ≤55%). Only two of these sequences (isolates 10613 and 10614), both isolated in Argentina in 1989, were very similar (identity of 82.7% and 95.8% at nucleotidic and peptidic level, respectively). Similar results were obtained when each third of the VP1 regions was used for alignment and phylogenetic relationship studies (data not shown).

A few CV-A11 and CV-A17 and several CV-A13 isolates obtained from Madagascar between 1994 and 2005, had already been characterized on the basis of their partial VP1 sequences (about 320 nt corresponding to the 3′ third of the gene) [13], [14], [21], [29].

To study further the diversity of the HEV-C viruses in Madagascar, these partial VP1 sequences of these new HEV-C isolates were compared to those of the 2002 HEV-C isolates.

Most of the newly introduced CV-A13 sequences from Madagascar (31 out of 37) fell into the cluster B supported by a bootstrap value of 98% (Figure 2). The other six sequences fell into cluster C (bootstrap value of 93%). In this cluster, the isolate IH-024/97 diverged slightly from the six other ones (nt identity ranging from 77.3% to 78.9%) that featured together a nt identity ≥91%.

The partial VP1 sequences of two CV-A11 and two CV-A17 isolates collected in Madagascar in 1997 [21] also fell into the clusters defined in these types by the Madagascan viruses isolated in 2002 (data not shown).

### Sequencing of full length genomes; analysis of recombination events

In order to characterize further the genomic features of the non-PV HEV-C lineages cocirculating in Madagascar in 2002, we selected 10 viruses as representatives of 10 different lineages [14]. Three were CV-A11, four were CV-A13 (three from the cluster B, one from the cluster C), two were CV-A17 and one was HEV-99.

Full-length genomic sequences were determined by gene walking. The genomes ranged from 7,444 to 7462 nt in length, encoding polyproteins of 2,210 to 2,214 aa. The 5′-UTR were 734 to 748 nt-long, the 3′-UTR were 71 to 72 nt-long (including the TAG stop codon). The polyprotein cleavage sites were identified by comparison with those of the prototype strains. All the genomes displayed the canonical organization of the enterovirus genomes and had no particular features differenciating them from reported full-length genomes of HEV of the same types.

Partial sequencing of different genomic regions of the Madagascan HEV-C had previously highlighted several intertypic recombinant lineages [14]. To decipher better the rules governing recombination between these HEV-C, we compared their full-length genomes by phylogenetic, bootscanning and similarity plot analyses. This study also included two viruses isolated in Madagascar in 2002 whose full-length sequence had been previously reported: MAD004 was a type 2 VDPV isolated from the stools of a patient with acute flaccid paralysis; CV-A17 67591 was a field isolate genetically related to MAD004 [14], [35].

**Figure 3:**
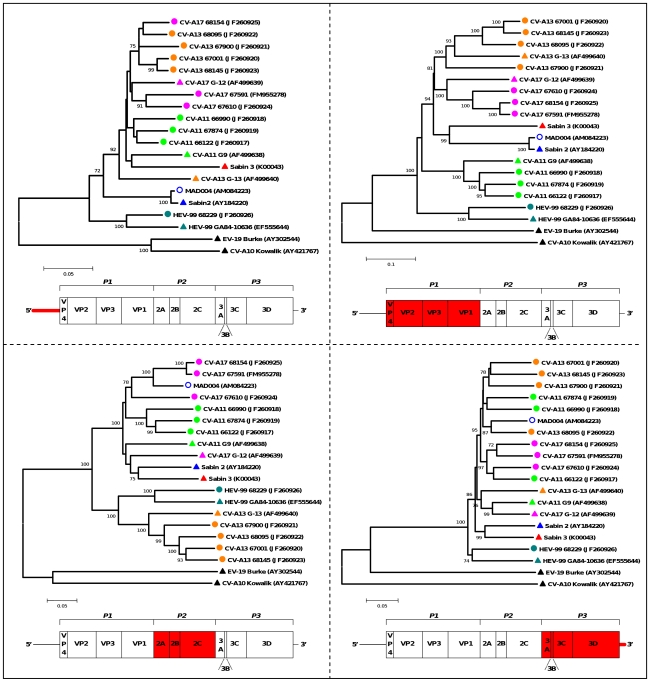
Phylograms depicting the relationships between the studied HEV-C in different genomic regions. The percentage of bootstrap replicates is indicated at nodes if higher than 70%. The length of branches is proportional to the number of nucleotide changes (percent divergence). The sequences of CV-A10 and EV-19 (members of HEV species A and B, respectively) were introduced for correct rooting of the tree. Triangles indicate the prototype strains, circles the field strains; the VDPV strain MAD004 is labelled with open circles. Each color corresponds to a given HEV-C serotype. Below each tree, the region taken in consideration for alignment is highlighted in red in the schematic diagram of the genome.

Phylogenetic trees were drawn from alignments of 5′-UTR, capsid, P2 and P3-3′-UTR sequences (Figure 3). Incongruences observed between the phylograms confirmed that several recombination events occurred to give rise to the field strain genomes. Interestingly, none of the field genome remained clustered with the prototype strain of its own type outside the capsid-encoding region P1, except isolate HEV-99 68229.

**Figure 4:**
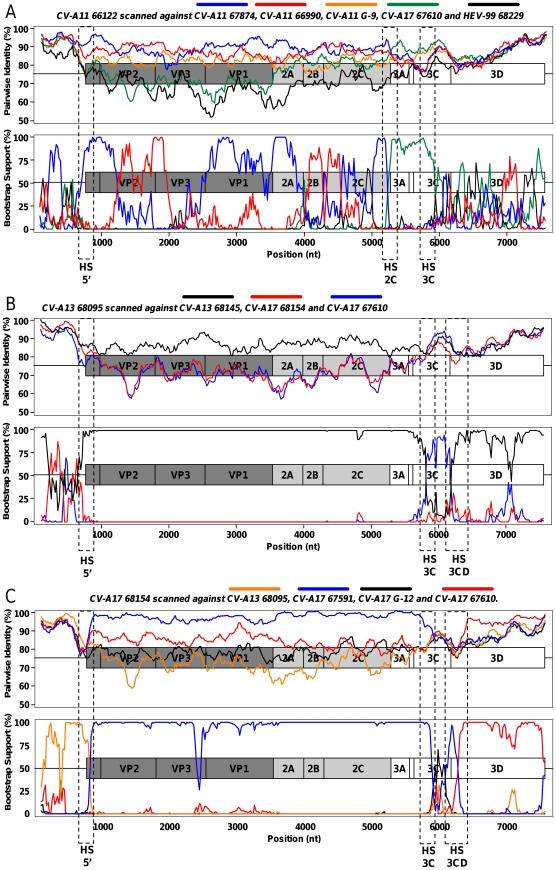
Pairwise comparison and bootscanning analysis of CV-A11 66122 (A), CV-A13 68095 (B) and CV-A17 68154 (C) full-length genomes. The dotted rectangles indicate the putative recombination hotspots.

**Figure 5:**
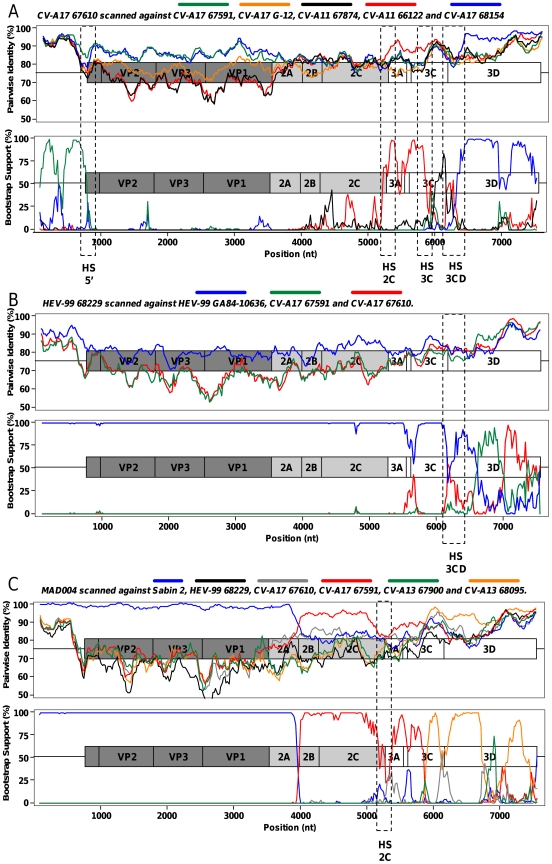
Pairwise comparison and bootscanning analysis of CV-A17 67610 (A), HEV-99 68229 (B) and MAD004 (C) full-length genomes. The dotted rectangles indicate the putative recombination hotspots.

To locate precisely breakpoints, similarity plots and bootscanning analyses were carried out. Each genome was compared with all the others; the curves showing particularly similar domains (high pairwise identities) and switches of domain clustering supported by high bootstrap values are shown in Figures 4 and 5.

CV-A11 genomes featured several recombination events (Figure 4A). The first part of the 66122 genome (5′-UTR, capsid and P2) comprised alternate regions most closely related to the isolates 66990 and 67874 (at least 5 switches were observed). The 5′ part of the P3 region of 66122 was most closely related to that of the isolate CV-A17 67610 (breakpoint located near the 2C/3A junction). Another recombination site was located in the 3C region but none of the studied strains was identified as the parental strain responsible for donating the 3D-3′-noncoding sequence of the 66122 genome. Through this analysis, the genome of the isolate 66122 appeared to result of at least eight intra- and intertypic recombination events.

CV-A13 68095 genome (Figure 4B) was very close to CV-A13 68145 in most parts (P1, P2 and most of the P3). However, it was closer to CV-A17 68154 in the 5′-UTR and to CV-A17 67610 in the 3C region.

Sequences of the CV-A17 isolates only clustered together in the P1 capsidic region. The isolates 68154 and 67591 were closely related from the P1 region to the 3C region (Figure 4C); Isolate 68154 was shown to be related to CV-A13 68095 in the 5′UTR and to CV-A17 67610 in the 3D region (Figure 4C).

Compared to the two other CV-A17 isolates, the strain 67610 was slightly divergent in the capsidic and P2 region but did not feature evidence of recombination. At least two recombination events were detected downstream (Figure 5A): from the end of the 2C region until the middle of the 3C region, this genome was closer to CV-A11 66122 than to the two other CV-A17 genomes; its 3D region was closer to the CV-A17 68154 one.

Pairwise comparison and bootscanning analysis of the HEV-99 68229 genome confirmed that most regions were not subjected to recombination (Figure 5B). The genome of this isolate remained close to that of the prototype GA84-10636 over its entire length, except in the 3D-3′-UTR region. In this region, CV-A17 67591 and 67610 were found to be the closest sequences among the studied ones.

The MAD004 genome had already been shown to associate sequences sharing common ancestors with Sabin 2, CV-A17 67591 and CV-13 68095 [14]. A Sabin 2/CV-A17 67591 breakpoint had been previously identified in the 2A-encoding region [35]. Our analyses showed that the structure of the non-structural sequence of MAD004 may be more complex than previously thought (Figure 5C). A putative 67610/68095 breakpoint was found in the 3C region but two genomic fragments (from the end of 2C through 3C and from the middle of 3D through the 3′ extremity) remained poorly determined, suggesting that additional recombination events involving unknown parental strains may have shaped the non-PV part of the MAD004 genome.

An overview of these results allowed us to identify four genomic regions where breakpoints were frequently observed (referred to as HS5′, HS2C, HS3C and HS3CD in Figures 4 and 5). These sites were found at the end of the 5′-UTR (4 hits), at the end of the 2C regions (3 hits), inside the 3C region (4 hits) and surrounding the 3C/3D junction (5 hits). These regions may constitute hot spots of recombination in HEV-C genomes.

## Discussion

Intraspecies recombination between enteroviruses is known to be very frequent and constitutes an important mechanism of evolution of these viruses. However, little is known about the frequency and rules governing genetic exchanges between co-circulating non-PV enteroviruses.

The panel of epidemiologically related HEV-C isolated from a small geographic area of Madagascar over the course of a few days [14] constitutes a precious tool to address the mechanisms shaping the HEV diversity.

Our analysis on VP1 sequences showed that the Madasgascan sequences defined clusters within their respective type. These clusters contained exclusively sequences of viruses isolated in Madagascar. This finding could be due to the geographic isolation of Madagascar, which could favour the appearance of topotypes in absence of introduction of strains from other countries. Interestingly, the clusters contained sequences from viruses isolated in different regions and years, indicating the active cocirculation of members of these groups through the island during several years. Nonetheless, as only a small number of sequences of field CV-A11, CV-A13, CV-A17 and HEV-99 isolates are available in public databases, the existence of putative Madagascan topotypes will need to be reassessed in the future, with respect to newly reported HEV-C from other countries.

Analyses of the full-length genomic sequences of some field strains isolated in 2002 in Madagascar refined our understanding about the relationship between these strains and highlighted the complexity of their genomes. Most appeared to have complex mosaic genomes constituted of fragments shared by other strains belonging to identical and/or different types. In most cases, the genetic exchanges concerned the 5′-UTR and the P3 regions.

Nevertheless, bootscan analysis suggested that recombination may occur inside the capsid region of CV-A11 genomes. Genetic recombination in the P1 region is thought to be rare, probably because of structural constraints that exist to properly assemble the viral capsid [7]. However, some cases of recombination within this region have been reported for HEV-A [36], [37], HEV-B [38], [39], [40] and circulating PV strains [19], [41], [42], [43], [44], [45].

Recombination in the P2 region did not appear to be frequent in this study, although recombination in the 2A region was often found in recombinant HEV, including VDPV [4], [7], [14], [35], [46].

Although only a few recombinant isolates were analyzed in detail, our results suggest that some genomic regions may be relatively prone to recombination, as many breakpoints were found near the 5′-UTR/V4 and 3C/3D junctions, at the end of 2C and within 3C. These regions may constitute hotspots for the recombination between HEV-C.

Several recombination hotspots have been proposed previously for HEV and other members of the family *Enterovirus*, particularly in the 2A region and near the 5′-UTR/VP4 junction [40], [47], [48], [49].

The existence of recombination hotspots may be accounted for by particular topological features of the RNA in these regions, facilitating the recombination process [50], [51], [52], [53]. Alternatively, hotspots in circulating recombinants may also result from the high fitness of viruses with genomes generated by recombination in particular regions, resulting in the disappearance of viruses with other genomic recombination features [54].

Interestingly, the HEV-99 isolates appeared to be poorly subjected to recombination with the other Madagascan HEV-C: in the previously published trees (Figure 4 in [14]), all the HEV-99 sequences grouped into a single cluster, regardless of the genomic region that was considered; this cluster did not contain any sequences from viruses belonging to other types.

The unique HEV-99 lineage may reflect the putative recent introduction of HEV-99 in the region from which the isolates were collected. A short period of circulation may have prevented HEV-99 isolates from evolving by recombination with other HEV-C isolates. A more likely hypothesis is that this HEV-99 lineage is poorly susceptible to recombination with CV-A11, CV-A13 and CV-A17. Support for this hypothesis is provided the high degree of similarity of the HEV-99 68229 sequence with the sequence of its prototype (HEV-99 GA84-10636) in most genomic regions, whereas this strain was isolated in Georgia, USA, in 1984 [32].

This observation is unusual for HEV since it is generally accepted that non-capsid regions of the HEV genomes evolve rapidly by recombination [4], [7]. If confirmed in other contexts, this low rate of recombination between HEV-99 strains and other HEV-C may be accounted for by functional incompatibility preventing exchanges of sequences between HEV-99 and other HEV-C. Such functional interactions affecting viral multiplication were recently reported in certain in vitro-made recombinant HEV-C [55], indicating that constraints can limit intertypic recombination.

In conclusion, most of the Madagascan genomes sequenced here appeared to be mosaic genomes. In particular, the CV-A11 66122 isolate had a genome composed of at least 9 fragments of different origins implicating many intra- and intertypic recombination events.

Such a high degree of complexity in a recombinant genome was previously reported for a Madagascan VDPV whose genome was constituted of fragments from non-PV HEV-C and 2 types of PV [13], [46]. The frequency and complexity of recombination in this dense HEV-C population strongly suggest that recombination is a widespread mechanism of genetic plasticity that generates shifts in biodiversity through the continual exchange of functional units between cocirculating genomes. The polioviruses appear to be the major pathogenic member of this HEV-C ecosystem, continually interacting through recombination with its HEV-C partners.

## References

[1] Racaniello VR, Knipe DM (2007). *Picornaviridae*: The Viruses and Their Replication.. Fields Virology.

[2] Picornaviridae study group website. http://www.picornaviridae.1com/sequences/sequences.htm.

[3] International Committee on Taxonomy of Viruses website. http://www.ictvdb.org/.

[4] Lukashev AN (2005). Role of recombination in evolution of enteroviruses.. Rev Med Virol.

[5] Lukashev AN (2010). Recombination among picornaviruses.. Rev Med Virol.

[6] Simmonds P (2006). Recombination and selection in the evolution of picornaviruses and other Mammalian positive-stranded RNA viruses.. J Virol.

[7] Simmonds P, Welch J (2006). Frequency and dynamics of recombination within different species of human enteroviruses.. J Virol.

[8] Savolainen-Kopra C, Blomqvist S (2010). Mechanisms of genetic variation in polioviruses.. Rev Med Virol.

[9] Minor P (2009). Vaccine-derived poliovirus (VDPV): Impact on poliomyelitis eradication.. Vaccine.

[10] Kew OM, Sutter RW, de Gourville EM, Dowdle WR, Pallansch MA (2005). Vaccine-derived polioviruses and the endgame strategy for global polio eradication.. Annu Rev Microbiol.

[11] Combelas N, Holmblat B, Joffret ML, Colbere-Garapin F, Delpeyroux F (2011). Recombination between Poliovirus and Coxsackie A Viruses of Species C: A Model of Viral Genetic Plasticity and Emergence.. Viruses.

[12] Zhang Y, Zhang F, Zhu S, Chen L, Yan D (2010). A Sabin 2-related poliovirus recombinant contains a homologous sequence of human enterovirus species C in the viral polymerase coding region.. Arch Virol.

[13] Rakoto-Andrianarivelo M, Gumede N, Jegouic S, Balanant J,  Andriamamonjy SN (2008). Reemergence of recombinant vaccine-derived poliovirus outbreak in Madagascar.. J Infect Dis.

[14] Rakoto-Andrianarivelo M, Guillot S, Iber J, Balanant J, Blondel B (2007). Co-circulation and evolution of polioviruses and species C enteroviruses in a district of Madagascar.. PLoS Pathog.

[15] Dedepsidis E, Kyriakopoulou Z, Pliaka V, Kottaridi C, Bolanaki E (2007). Retrospective characterization of a vaccine-derived poliovirus type 1 isolate from sewage in Greece.. Appl Environ Microbiol.

[16] Adu F, Iber J, Bukbuk D, Gumede N, Yang SJ (2007). Isolation of recombinant type 2 vaccine-derived poliovirus (VDPV) from a Nigerian child.. Virus Res.

[17] Anonymous (2001). Acute flaccid paralysis associated with circulating vaccine-derived poliovirus--Philippines, 2001.. MMWR Morb Mortal Wkly Rep.

[18] Kew O, Morris-Glasgow V, Landaverde M, Burns C, Shaw J (2002). Outbreak of poliomyelitis in Hispaniola associated with circulating type 1 vaccine-derived poliovirus.. Science.

[19] Liu HM, Zheng DP, Zhang LB, Oberste MS, Pallansch MA (2000). Molecular evolution of a type 1 wild-vaccine poliovirus recombinant during widespread circulation in China.. J Virol.

[20] Rousset D, Rakoto-Andrianarivelo M, Razafindratsimandresy R, Randriamanalina B, Guillot S (2003). Recombinant vaccine-derived poliovirus in Madagascar.. Emerg Infect Dis.

[21] Rakoto-Andrianarivelo M, Rousset D, Razafindratsimandresy R, Chevaliez S, Guillot S (2005). High frequency of human enterovirus species C circulation in Madagascar.. J Clin Microbiol.

[22] Bessaud M, Jegouic S, Joffret ML, Barge C, Balanant J (2008). Characterization of the genome of human enteroviruses: design of generic primers for amplification and sequencing of different regions of the viral genome.. J Virol Methods.

[23] Tamura K, Dudley J, Nei M, Kumar S (2007). MEGA4: Molecular Evolutionary Genetics Analysis (MEGA) software version 4.0.. Mol Biol Evol.

[24] Martin DP, Posada D, Crandall KA, Williamson C (2005). A modified bootscan algorithm for automated identification of recombinant sequences and recombination breakpoints.. AIDS Res Hum Retroviruses.

[25] Lole KS, Bollinger RC, Paranjape RS, Gadkari D, Kulkarni SS (1999). Full-length human immunodeficiency virus type 1 genomes from subtype C-infected seroconverters in India, with evidence of intersubtype recombination.. J Virol.

[26] Hamming RW (1950). Error Detecting and Error Correcting Codes.. Bell System Tech Journal.

[27] Kimura M (1980). A simple method for estimating evolutionary rates of base substitutions through comparative studies of nucleotide sequences.. J Mol Evol.

[28] Oberste MS, Maher K, Kilpatrick DR, Pallansch MA (1999). Molecular evolution of the human enteroviruses: correlation of serotype with VP1 sequence and application to picornavirus classification.. J Virol.

[29] Caro V, Guillot S, Delpeyroux F, Crainic R (2001). Molecular strategy for ‘serotyping’ of human enteroviruses.. J Gen Virol.

[30] Oberste MS, Maher K, Kilpatrick DR, Flemister MR, Brown BA (1999). Typing of human enteroviruses by partial sequencing of VP1.. J Clin Microbiol.

[31] Nix WA, Oberste MS, Pallansch MA (2006). Sensitive, seminested PCR amplification of VP1 sequences for direct identification of all enterovirus serotypes from original clinical specimens.. J Clin Microbiol.

[32] Brown BA, Maher K, Flemister MR, Naraghi-Arani P, Uddin M (2009). Resolving ambiguities in genetic typing of human enterovirus species C clinical isolates and identification of enterovirus 96, 99 and 102.. J Gen Virol.

[33] Arita M, Zhu SL, Yoshida H, Yoneyama T, Miyamura T (2005). A Sabin 3-derived poliovirus recombinant contained a sequence homologous with indigenous human enterovirus species C in the viral polymerase coding region.. J Virol.

[34] Junttila N, Leveque N, Kabue JP, Cartet G, Mushiya F (2007). New enteroviruses, EV-93 and EV-94, associated with acute flaccid paralysis in the Democratic Republic of the Congo.. J Med Virol.

[35] Jegouic S, Joffret ML, Blanchard C, Riquet FB, Perret C (2009). Recombination between polioviruses and co-circulating Coxsackie A viruses: role in the emergence of pathogenic vaccine-derived polioviruses.. PLoS Pathog.

[36] Huang SW, Hsu YW, Smith DJ, Kiang D, Tsai HP (2009). Reemergence of enterovirus 71 in 2008 in taiwan: dynamics of genetic and antigenic evolution from 1998 to 2008.. J Clin Microbiol.

[37] Lin KH, Hwang KP, Ke GM, Wang CF, Ke LY (2006). Evolution of EV71 genogroup in Taiwan from 1998 to 2005: an emerging of subgenogroup C4 of EV71.. J Med Virol.

[38] Oberste MS, Penaranda S, Pallansch MA (2004). RNA recombination plays a major role in genomic change during circulation of coxsackie B viruses.. J Virol.

[39] Bouslama L, Nasri D, Chollet L, Belguith K, Bourlet T (2007). Natural recombination event within the capsid genomic region leading to a chimeric strain of human enterovirus B. J Virol.

[40] Lukashev AN, Lashkevich VA, Ivanova OE, Koroleva GA, Hinkkanen AE (2005). Recombination in circulating Human enterovirus B: independent evolution of structural and non-structural genome regions.. J Gen Virol.

[41] Blomqvist S, Savolainen-Kopra C, Paananen A, El Bassioni L, El Maamoon Nasr EM (2010). Recurrent isolation of poliovirus 3 strains with chimeric capsid protein Vp1 suggests a recombination hot-spot site in Vp1.. Virus Res.

[42] Martin J, Samoilovich E, Dunn G, Lackenby A, Feldman E (2002). Isolation of an intertypic poliovirus capsid recombinant from a child with vaccine-associated paralytic poliomyelitis.. J Virol.

[43] Dedepsidis E, Pliaka V, Kyriakopoulou Z, Brakoulias C, Levidiotou-Stefanou S (2008). Complete genomic characterization of an intertypic Sabin 3/Sabin 2 capsid recombinant.. FEMS Immunol Med Microbiol.

[44] Blomqvist S, Bruu AL, Stenvik M, Hovi T (2003). Characterization of a recombinant type 3/type 2 poliovirus isolated from a healthy vaccinee and containing a chimeric capsid protein VP1.. J Gen Virol.

[45] Mueller JE, Bessaud M, Huang QS, Martinez LC, Barril PA (2009). Environmental poliovirus surveillance during oral poliovirus vaccine and inactivated poliovirus vaccine use in Cordoba Province, Argentina.. Appl Environ Microbiol.

[46] Joffret ML, Jegouic S, Bessaud M, Balanant J, Tran C Common and diverse features of co-circulating type 2 and 3 recombinant vaccine-derived polioviruses isolated from patients with poliomyelitis and healthy children.. J Infect Dis in press.

[47] Santti J, Hyypia T, Kinnunen L, Salminen M (1999). Evidence of recombination among enteroviruses.. J Virol.

[48] Huang T, Wang W, Bessaud M, Ren P, Sheng J (2009). Evidence of recombination and genetic diversity in human rhinoviruses in children with acute respiratory infection.. PLoS One.

[49] Wisdom A, Kutkowska AE, McWilliam Leitch EC, Gaunt E, Templeton K (2009). Genetics, recombination and clinical features of human rhinovirus species C (HRV-C) infections; interactions of HRV-C with other respiratory viruses.. PLoS One.

[50] Romanova LI, Blinov VM, Tolskaya EA, Viktorova EG, Kolesnikova MS (1986). The primary structure of crossover regions of intertypic poliovirus recombinants: a model of recombination between RNA genomes.. Virology.

[51] Dedepsidis E, Kyriakopoulou Z, Pliaka V, Markoulatos P (2010). Correlation between recombination junctions and RNA secondary structure elements in poliovirus Sabin strains.. Virus Genes.

[52] Kirkegaard K, Baltimore D (1986). The mechanism of RNA recombination in poliovirus.. Cell.

[53] Tolskaya EA, Romanova LI, Blinov VM, Viktorova EG, Sinyakov AN (1987). Studies on the recombination between RNA genomes of poliovirus: the primary structure and nonrandom distribution of crossover regions in the genomes of intertypic poliovirus recombinants.. Virology.

[54] Worobey M, Holmes EC (1999). Evolutionary aspects of recombination in RNA viruses.. J Gen Virol.

[55] Liu Y, Wang C, Mueller S, Paul AV, Wimmer E (2010). Direct interaction between two viral proteins, the nonstructural protein 2C and the capsid protein VP3, is required for enterovirus morphogenesis.. PLoS Pathog.

